# Parenting and the Adjustment of Children Born to Gay Fathers Through Surrogacy

**DOI:** 10.1111/cdev.12728

**Published:** 2017-01-23

**Authors:** Susan Golombok, Lucy Blake, Jenna Slutsky, Elizabeth Raffanello, Gabriela D. Roman, Anke Ehrhardt

**Affiliations:** ^1^ University of Cambridge; ^2^ Columbia University

## Abstract

Findings are presented on a study of 40 gay father families created through surrogacy and a comparison group of 55 lesbian mother families created through donor insemination with a child aged 3–9 years. Standardized interview, observational and questionnaire measures of stigmatization, quality of parent–child relationships, and children's adjustment were administered to parents, children, and teachers. Children in both family types showed high levels of adjustment with lower levels of children's internalizing problems reported by gay fathers. Irrespective of family type, children whose parents perceived greater stigmatization and children who experienced higher levels of negative parenting showed higher levels of parent‐reported externalizing problems. The findings contribute to theoretical understanding of the role of family structure and family processes in child adjustment.

Research on children with same‐sex parents was initiated in the 1970s to inform custody cases involving a lesbian mother. Since that time, longitudinal studies have followed up children of lesbian mothers to adulthood, investigations have been conducted on children raised in lesbian mother families from birth, data have been obtained from general population samples of lesbian mother families, and meta‐analyses of these studies have been carried out (for reviews, see Fedewa, Black, & Ahn, 2014; Goldberg, 2010; Patterson, 2006, 2009). This substantial body of research has consistently shown that children in lesbian mother families do not differ from children in comparable groups of heterosexual parent families in terms of psychological adjustment. Instead, difficulties experienced by these children appear to be associated with stigmatization by the outside world (Bos & Gartrell, 2010; Bos & van Balen, 2008).

The circumstances of children with gay fathers are somewhat different from those of children with lesbian mothers in that it is unusual for fathers, whether heterosexual or gay, to be primary caregivers. Although research on fathering has shown that the constructs of fathering and mothering involving positive engagement, warmth, and responsiveness are largely the same and that the processes through which heterosexual fathers influence their children are similar to that of mothers (for a review, see Fagan, Day, Lamb, & Cabrera, 2014), it is widely assumed that fathers are less suited to parenting than are mothers (Biblarz & Stacey, 2010). Moreover, children with gay fathers may be exposed to greater stigmatization than children with lesbian mothers because gay father families possess the additional nontraditional feature of being headed solely by men (Golombok & Tasker, 2010). Similarly, gay fathers may themselves be exposed to greater stigmatization regarding their sexual identity than are lesbian mothers (Goldberg & Smith, 2011).

Controlled, in‐depth studies of children of gay fathers were initiated following the millennium and largely focused on gay father families formed through adoption. In a study of the psychological adjustment of 2‐year‐olds (Goldberg & Smith, 2013), no differences were found between children with gay, lesbian, and heterosexual adoptive parents. However, parental depression, relationship conflict, and lack of preparation for the adoption were associated with children's emotional and behavioral problems. Farr, Forssell, and Patterson (2010a, 2010b) found preschool children adopted in infancy by gay fathers to be as well adjusted as those adopted by lesbian or heterosexual parents. In an observational assessment of family play, the gay couples were rated not only as less supportive of each other but also as less undermining than were the heterosexual couples (Farr & Patterson, 2013). A comparison of adoptive gay father families, adoptive lesbian mother families, and adoptive heterosexual parent families with 3‐ to 9‐year‐old children was conducted in the United Kingdom (Golombok et al., 2014). Where differences were identified between family types, these indicated more positive parental well‐being and parent–child relationships and lower levels of children's externalizing problems in gay father families compared to heterosexual parent families.

Although limited, research on adoptive gay father families indicates that children can flourish in this family environment. However, gay father families with children born through surrogacy differ not only from the traditional family with respect to the sexual orientation and gender of the parents but also from adoptive gay father families in that the children have both a genetic and nongenetic father as well as a genetic mother (the egg donor) and a gestational mother (the surrogate). A longitudinal study of children in heterosexual families created through surrogacy found high levels of psychological adjustment in surrogacy children in the preschool years (Golombok, MacCallum, Murray, Lycett, & Jadva, 2006a; Golombok et al., 2006b; Golombok, Murray, Jadva, MacCallum, & Lycett, 2004) but raised levels of emotional and behavioral problems at age 7 (Golombok et al., 2011), the age at which children acquire an understanding of biological inheritance and the biological concept of family (Gregg, Solomon, Johnson, Zaitchik, & Carey, 1996; Williams & Smith, 2010) and of the meaning and implications of the absence of a biological connection to parents (Brodzinsky, 2011). Raised levels of emotional and behavioral problems among the surrogacy children were no longer apparent at age 10 (Golombok, Blake, Casey, Roman, & Jadva, 2013) or age 14 (Golombok, Ilioi, Blake, Roman, & Jadva, 2016).

Although there has been a dramatic rise in the number of gay men having children through surrogacy (Berkowitz, 2013), the creation of gay father families through assisted reproductive technologies is such a recent phenomenon that there is little research on children born in this way. In an uncontrolled, questionnaire‐based study of 68 gay father families with 3‐ to 10‐year‐old children born through gestational surrogacy, the children of gay fathers were reported to show significantly lower levels of adjustment problems compared to data obtained from general population norms, with the daughters of gay fathers appearing to exhibit particularly low levels of internalizing problems (Green, Rubio, Bergman, & Katuzny, 2015). In a questionnaire‐based study in Italy, gay father families formed through surrogacy did not differ from lesbian mother families formed through donor insemination or heterosexual parent families with naturally conceived children with respect to parent‐reported family functioning or the emotional regulation or adjustment of children aged around 4 years (Baiocco et al., 2015).

The aim of the present investigation was to conduct a controlled, in‐depth study of gay father families created through surrogacy with children who were old enough to understand that their family structure differed from that of other children. The study focused on families with children aged at least 3 years, as it is not until age 3 that adopted children acquire a rudimentary understanding of having been born into a different family (Brodzinsky, 2011), and children in single‐parent families become aware that their family differs from the traditional family with a mother and a father (Zadeh, Freeman, & Golombok, 2016). The upper age limit of age 9 was chosen to optimize the sample size of this emerging family form while ensuring the appropriateness of the measures across the age range.

From a theoretical perspective, the study was grounded in a developmental contextual systems approach (Overton, 2015), whereby bidirectional relations between the children, the family, and the wider social world are viewed as influential in development. The study tested the hypothesis that gay father families created through surrogacy would experience greater difficulties in terms of stigmatization, parenting, and child adjustment than a comparison group of lesbian mother families created through donor insemination due to the additional challenges faced by gay father families formed in this way. Although adoptive gay father families have not been found to show elevated levels of problems compared to adoptive lesbian mother families or adoptive heterosexual parent families, greater difficulties were predicted for gay father families formed through surrogacy as raised levels of psychological problems have previously been found among early school‐age children born to heterosexual parents through surrogacy (Golombok et al., 2011). Moreover, gay father families formed through surrogacy may face greater stigmatization than adoptive gay father families resulting from their use of a surrogate and an egg donor to create a family. Lesbian mother families formed through donor insemination were chosen as the comparison group to control for both the nonheterosexual orientation of the parents and the use of third‐party assisted reproduction, and because of the large body of research showing that children with lesbian mothers do not differ in psychological adjustment from children with heterosexual parents. It was also hypothesized, based on the growing body of research showing that parental sexual orientation is less predictive of child adjustment than is the quality of family relationships (e.g., Bos & Gartrell, 2010; Chan, Raboy, & Patterson, 1998; Farr et al., 2010a, 2010b; Golombok et al., 2014), that stigmatization of the family and quality of parenting would be more strongly associated with children's adjustment than would family type.

## Method

### Participants

Forty gay father families created through surrogacy and a comparison group of 55 lesbian mother families created through donor insemination participated in the study in the United States. As this is the first in‐depth study of children born to gay fathers through surrogacy, it was necessary to rely on a volunteer sample of this small and hard‐to‐reach population. Thus, the gay father families were recruited through surrogacy agencies that specialized in working with gay men, gay father social groups, and snowballing. The lesbian mother families were similarly recruited through the Donor Sibling Registry, lesbian mother social groups, and snowballing. The inclusion criteria for both the gay father families and lesbian mother families were that the couple had a child aged between 3 and 9 years, and had lived together since the child's birth.

There was no significant difference between family types in the age of the target child, *F*(1, 93) = 0.04, *p* = .82, with the average age being 5.3 years, or with respect to the child's gender, χ^2^(1) = 0.77, *p* = .25. The age of the parents differed significantly between family types, *F*(1, 93) = 47.43, *p *<* *.001, reflecting the older age of the gay fathers (average age 47 years) than the lesbian mothers (average age 40 years). There was no difference between family types in the marital status of the parents, χ^2^(1) = 1.49, *p* = .19, or in the number of siblings in the family, χ^2^(2) = .51, *p* = .77. There was a significant difference between family types in household income, χ^2^(3) = 49.71, *p* *<* .001, reflecting a higher income in gay father families. In families with more than one child in the required age range, the oldest was selected.

### Procedure

The majority of families were assessed at home. However, 35% of gay father families and 42% of lesbian mother families were assessed by Skype because of the geographical distance from the researchers. Written informed consent to participate in the investigation was obtained from each parent. Ethical approval was granted by the University of Cambridge Psychology Research Ethics Committee and the New York State Psychiatric Institutional Review Board. Each parent was administered an audio‐recorded standardized interview that lasted approximately 1.5 hr, a video‐recorded observational assessment of parent–child interaction, and standardized questionnaires. Teachers were administered a questionnaire. Data were collected between September 2013 and December 2015.

### Measures

#### Quality of Parenting

Each parent was interviewed using an adaptation of a semistructured interview designed to assess quality of parenting that has been validated against observational ratings of mother–child relationships in the home (Quinton & Rutter, 1988) and has been used successfully in previous studies of same‐sex parent families with children of the same age (Golombok et al., 2014). Detailed accounts are obtained of the child's behavior and the parent's response to it, with particular reference to interactions relating to warmth and control. A flexible style of questioning is used to elicit sufficient information for each variable to be rated by trained researchers using a standardized coding scheme based on a detailed coding manual. Thus, ratings are carried out by the researchers using in‐depth information obtained from the parents.

The following variables were coded: (a) *expressed warmth* from 0 (*none*) to 5 (*high*) took account of the parent's tone of voice, facial expressions, and gestures in addition to what the parent said about the child; (b) *sensitive responding* from 1 (*low*) to 4 (*high*) represented the parent's ability to recognize and respond appropriately to the child's needs; (c) *quality of interaction* from 1 (*poor*) to 4 (*very good*) was based on the extent to which the parent and child wanted to be with each other and enjoyed each other's company; (d) *frequency of battles* from 1 (*never/rarely*) to 6 (*few times daily*) assessed the frequency of parent–child conflict; (e) *level of battles* from 0 (*none*) to 3 (*major*) assessed the severity of parent–child conflict; and (f) *disciplinary aggression* from 0 (*none*) to 2 (*moderate*) assessed the level of anger shown by the parent toward the child. To establish interrater reliability, 30 randomly selected interviews were coded by a second rater. The intraclass correlations for expressed warmth, sensitive responding, frequency of battles, and disciplinary aggression were 0.77, 0.73, 1.0, and 0.8, respectively. It was not appropriate to calculate intraclass correlations for quality of interaction and level of battles as they operated almost as binary variables. However, the percentage agreement between raters for these variables was 94% and 100%, respectively. Total scores of positive parenting (expressed warmth, sensitive responding, and quality of interaction) and negative parenting (frequency of battles, severity of battles, and disciplinary aggression) were computed for each parent using principal component analysis according to the procedure outlined in Golombok et al. (2013). Higher scores reflected more positive parenting (e.g., enthusiasm about the child, recognition of the child's worries, and enjoyment of the child's company) and more negative parenting (e.g., a high frequency and severity of conflict, loss of temper, and physical aggression), respectively. The factors explained over 46% of variance in the items and all of the factor loadings were above .55. The correlation between the positive parenting factor and the negative parenting factor was −.317.

#### Parent–Child Interaction

Within each family, each parent–child dyad participated in an observational assessment of parent–child interaction. In order to avoid practice effects, the Etch‐A‐Sketch task (Stevenson‐Hinde & Shouldice, 1995) was used with the parent who spent most time with the child, and the Co‐Construction task (Steele et al., 2007) was used with the other parent. In the one third of families where both parents shared parenting equally, the tasks were randomly assigned. The Etch‐A‐Sketch is a drawing tool with two dials that allow one person to draw vertically and the other to draw horizontally. The parent and child were asked to copy a picture of a house, each using one dial only, with clear instructions not to use the other dial. With the Co‐Construction task, the parent and child were given a set of wooden building blocks and instructed to build something together using as many blocks as possible. The Etch‐A‐Sketch and Co‐Construction sessions were video recorded and coded using the Parent–Child Interaction System (PARCHISY; Deater‐Deckard & Petrill, 2004) to assess the construct of mutuality, that is, the extent to which the parent and child engaged in positive dyadic interaction characterized by warmth, mutual responsiveness, and cooperation. The following variables were rated on a 7‐point scale ranging from 1 (*no instances*) to 7 (*constant, throughout interaction*): (a) *child's responsiveness to parent* assessed the extent to which the child responded immediately and contingently to the parent's comments, questions, or behaviors; (b) *parent's responsiveness to child* assessed the extent to which the parent responded immediately and contingently to the child's comments, questions, or behaviors; (c) *dyadic reciprocity* assessed the degree to which the dyad showed shared positive affect, eye contact, and a “turn‐taking” quality of interaction; and (d) *dyadic cooperation* assessed the degree of agreement about whether and how to proceed with the task. To establish interrater reliability, 50 randomly selected video recordings were coded by a second rater. The intraclass correlations for parent's responsiveness to child, child's responsiveness to parent, dyadic reciprocity, and dyadic cooperation were .92, .83, .75, and .85, respectively.

#### Perceived Stigma

Perceived stigma was measured using the personalized stigma subscale of a measure originally developed by Berger, Ferrans, and Lashley (2001) to assess HIV‐related stigma and later modified for the assessment of stigma associated with being gay (Frost, Parsons, & Nanin, 2007). The personalized stigma subscale comprises 10 items relating to negative social consequences associated with being gay. A total score is produced, with higher scores representing more negative experiences. The scale has been shown to have high internal consistency (Cronbach's alpha = .90) as well as construct validity (Frost et al., 2007). Cronbach's alpha for the present sample was .91.

#### Children's Adjustment

The presence of children's emotional and behavioral difficulties was assessed with the Strengths and Difficulties Questionnaire (SDQ; Goodman, 1994, 1997) administered to each parent to produce total scores of internalizing problems and externalizing problems (Goodman, Lamping, & Ploubidis, 2010), with higher scores indicating greater problems. The cutoff points for clinical problems are 9 for internalizing problems and 11 for externalizing problems. An independent assessment of the children's psychological adjustment was obtained by administering the SDQ to teachers. The cutoff point for both internalizing problems and externalizing problems is 11 for the teachers’ SDQ. Following permission from the parents, the questionnaire was mailed to the child's teacher with an enclosed stamped addressed envelope for return to the researcher. Teachers were informed that their responses to the questionnaire would not be reported back to the child's family or school. Questionnaires were received by 48 (50.5%) of the teachers. The SDQ has been shown to have good internal consistency, test–retest and interrater reliability, and concurrent and discriminative validity (Goodman, 1994, 1997). In a review of the reliability and validity of the SDQ based on 48 studies involving more than 130,000 children, Stone, Otten, Engels, Vermulst, and Janssens (2010) found the psychometric properties of the SDQ to be strong. Cronbach's alpha for the present sample was .71.

The children's externalizing and internalizing problems were also assessed during the interview with the parent who spent most time with the child or a parent selected at random in the families in which parenting was shared equally using a standardized procedure (Rutter, Cox, Tupling, Berger, & Yule, 1975). Detailed descriptions were obtained of any emotional or behavioral problems shown by the child. These descriptions of actual behavior, which included information about where the behavior was shown, severity of the behavior, frequency, precipitants, and course of the behavior over the past year, were transcribed and rated by a child psychiatrist who was unaware of the nature of the study. A high level of reliability (*r* = .85) between ratings made by social scientists and those made “blindly” by a child psychiatrist has been demonstrated for this procedure, and validity has been established through a high level of agreement with parents’ assessments of whether or not their children had emotional or behavioral difficulties (Rutter et al., 1975). Externalizing and internalizing problems were rated according to severity on a 3‐point scale ranging from 0 (*no disorder*) through 1 (*dubious or trivial disorder*) to 2 (*definite disorder*). Descriptive statistics are presented in Table 1.

**Table 1:** Descriptive Statistics for the Study Measures, Presented as Average Scores Across Both Parents

	Full sample	Lesbian mother families	Gay father families
	*n* (%)	*n* (%)	*n* (%)
Child's gender			
Boys	52 (54.7)	28 (50.9)	24 (60.0)
Girls	43 (45.3)	27 (49.1)	16 (40.0)
Number of siblings			
0	28 (29.5)	17 (30.9)	11 (27.5)
1	51 (53.7)	30 (54.5)	21 (52.5)
2 or more	16 (16.8)	8 (14.5)	8 (20.0)
Household income			
Less than $60K	13 (13.7)	13 (13.7)	0 (0)
Between $60K and 150K	35 (36.8)	31 (32.6)	4 (4.2)
Between $151K and 499K	29 (30.5)	10 (10.5)	19 (20.0)
More than $500K	18 (18.9)	1 (1.1)	17 (17.9)
	*M* (*SD*)	*M* (*SD*)	*M* (*SD*)
Child's age in months	68.31 (24.64)	67.84 (23.76)	68.95 (26.09)
Parent's age	43.10 (6.18)	40.05 (4.80)	47.29 (5.39)
Parent's educational level	4.68 (1.02)	4.45 (0.98)	5.01 (1.00)
Perceived stigma	15.53 (4.20)	15.89 (4.49)	14.94 (3.65)
Quality of parenting			
Positive parenting	−0.017 (0.87)	0.11 (0.85)	−0.21 (0.87)
Negative parenting	−0.001 (0.80)	−0.02 (0.76)	0.02 (0.85)
Observational assessment			
Parent responsiveness	4.78 (0.90)	4.98 (0.81)	4.50 (0.95)
Child responsiveness	4.59 (0.98)	4.77 (0.92)	4.33 (1.04)
Dyadic reciprocity	1.90 (0.78)	2.01 (0.78)	1.75 (0.79)
Dyadic cooperation	3.06 (1.18)	3.24 (1.15)	2.80 (1.20)
Parent‐rated SDQ			
Externalizing problems	4.02 (2.43)	3.94 (2.42)	4.14 (2.49)
Internalizing problems	2.75 (2.35)	3.30 (2.47)	1.86 (1.86)
Teacher‐rated SDQ			
Externalizing problems	3.88 (3.97)	3.45 (4.02)	4.53 (3.91)
Internalizing problems	2.00 (2.48)	2.38 (2.62)	1.42 (2.16)

Note

SDQ = Strengths and Difficulties Questionnaire.

#### Analysis Plan

The two research questions relating to family structure differences and factors associated with child adjustment were tested using multilevel modeling. This procedure is particularly useful when researching dyads that can be considered indistinguishable, as is the case for same‐sex parents (Smith, Sayer, & Goldberg, 2013). The variance of each variable is partitioned into variance occurring *within* families (i.e., the extent to which variation is due to differences between the two parents within a dyad; Level 1) and variance occurring *between* families (i.e., the extent to which variation is due to differences between families; Level 2). The variables that were measured separately for each parent, which were measured at Level 1, were modeled as random intercepts at Level 2, and represented average levels for each family. These random intercepts were used as outcome or predictor variables in regression models specified at Level 2, as the focus of the analyses was to identify differences between family types.

The hypothesis that gay father families would experience greater difficulties than lesbian mother families in terms of stigmatization, parent–child relationships, and child adjustment was tested using simple linear regression at Level 2, where models were specified separately for each outcome variable. The outcome variables were perceived stigma, positive and negative parenting, parent–child interaction (parent responsiveness, child responsiveness, dyadic reciprocity, and dyadic cooperation), and children's externalizing and internalizing problems as assessed by the parent‐rated SDQ and the teacher‐rated SDQ. Although the gay fathers were significantly older and economically better off than the lesbian mothers, parental age and family income were not related to the outcome variables (except for a significant relationship between income and both parent‐reported and teacher‐reported internalizing problems) and were therefore not included as control variables. The predictor in each model was family type, with lesbian mother families used as the reference group.

## Results

### Comparisons Between Gay Father and Lesbian Mother Families

Children in gay father families were reported by their parents to show significantly lower levels of internalizing problems than children in lesbian mother families (see Table 2). An alternative model, whereby the predictor was family income rather than family type, suggested a similar association between higher income and lower internalizing problems as reported by parents (intercept = 2.730, standardized slope = −.279, *p* = .004). The introduction of family type and family income as simultaneous predictors of internalizing problems lead to both effects becoming nonsignificant due to multicollinearity between the two constructs (standardized *r* = .68, *p* < .001). To understand whether the key predictor of internalizing problems was family type or family income, a multiple‐group multilevel model was specified, whereby the indicator of internalizing problems was regressed onto family income at Level 2 and the model run separately for each family type. Higher income did not predict lower internalizing problems in either lesbian mother or gay father families (standardized slope_lesbian_ = −.108, *p* = .498; standardized slope_gay_ = −.187, *p* = .358), suggesting that the key predictor of lower internalizing problems as reported by parents was family type rather than family income. However, in a further regression in which children's internalizing scores were residualized by income, family type did not explain further variance in children's internalizing problems. When the analysis was conducted using teachers’ scores, children's internalizing problems did not differ between family types.

**Table 2:** Differences in Parenting, Stigma, and Child Adjustment By Family Type

Outcome variable	Predictor	Coefficient	*p*	Standardized coefficient	*p*
Parent age	Intercept	40.055			
	Gay	7.233	.000	.649	.000
Family income	Intercept	1.982			
	Gay	1.343	.000	.699	.000
Positive parenting factor	Intercept	0.110			
	Gay	−0.244	.179	−.168	.180
Negative parenting factor	Intercept	0.002			
	Gay	−0.005	.976	−.005	.976
Parent responsiveness	Intercept	4.996			
	Gay	−0.343	.077	−.410	.173
Child responsiveness	Intercept	4.770			
	Gay	−0.429	.057	−.378	.037
Dyadic reciprocity	Intercept	2.011			
	Gay	−0.224	.209	−.195	.209
Dyadic cooperation	Intercept	3.168			
	Gay	−0.358	.181	−.317	.184
Perceived stigma	Intercept	15.853			
	Gay	−1.277	.126	−.258	.141
Parent‐rated SDQ (externalizing problems)	Intercept	3.915			
	Gay	0.483	.368	.108	.360
Parent‐rated SDQ (internalizing problems)	Intercept	3.216			
	Gay	−1.179	.014	−.267	.015
Teacher‐rated SDQ (externalizing problems)	Intercept	3.448			
	Gay	1.078	.344	.134	.358
Teacher‐rated SDQ (internalizing problems)	Intercept	2.379			
	Gay	−0.958	.159	−.191	.148

Note

Intercept = the overall level of the outcome variable in lesbian mother families; Gay = how much the score differed between gay father families and lesbian mother families; Coefficient = unstandardized coefficients; SDQ = Strengths and Difficulties Questionnaire.

There were no differences between gay father families and lesbian mother families in terms of perceived stigma, quality of parenting, parent–child interaction, or children's externalizing problems as reported by parents and teachers, with scores on the individual variables reflecting low levels of perceived stigma, high levels of positive parenting, low levels of negative parenting, average levels of parent–child interaction, and low levels of externalizing problems.

With respect to the ratings by the child psychiatrist, only 2 (5%) children in gay father families showed a definite disorder (1 with internalizing problems and 1 with externalizing problems) and only 2 (3.6%) children in lesbian mother families showed a definite disorder (1 with internalizing problems and 1 with externalizing problems). There was no difference between gay father and lesbian mother families in the proportion of children with a psychiatric disorder as rated by a child psychiatrist, χ^2^(1) = 0.11, *p* *=* .74.

### Stigma, Parenting, and Child Adjustment

To examine factors associated with children's adjustment, the variables of perceived stigma, positive parenting, negative parenting, and the four observational measures of parent–child interaction (parent responsiveness, child responsiveness, dyadic reciprocity, and dyadic cooperation) were entered into a Level 2 regression as predictors of children's externalizing and internalizing problems as reported by parents (one model per outcome).

With respect to externalizing problems, positive parenting and the four observational measures of parent–child interaction showed no significant effects and were therefore excluded from the model. The two remaining predictors were significant. Parents who perceived higher levels of stigma reported that their children showed higher levels of externalizing problems (estimate = 0.767, *SE* = 0.289, standardized *z *=* *2.638, *p* = .008), and children exposed to higher levels of negative parenting were reported by their parents to show higher levels of externalizing problems (estimate = 5.285, *SE* = 1.335, standardized *z *=* *4.277, *p* < .001). These effects could not have arisen due to multicollinearity, as the two predictors were not significantly related to each other. Regarding internalizing problems, none of the predictors was significant (*p* > .528).

The analyses were repeated using teacher‐reported externalizing and internalizing problems. None of the paths was significant.

## Discussion

Contrary to the hypothesis that children with gay fathers would show higher levels of adjustment difficulties than children with lesbian mothers, the children in both family types were reported by parents and teachers to show low levels of behavioral and emotional problems, and significantly lower levels of parent‐reported internalizing problems were found for the children of gay fathers than for the children of lesbian mothers. It is important to emphasize that children's internalizing problems were very low in both family types in relation to the cutoff point for clinical problems. The significant difference between family types reflected a difference between low levels of internalizing problems reported by lesbian mothers and even lower levels of internalizing problems reported by gay fathers.

There were no differences between the children of gay fathers and lesbian mothers in terms of externalizing problems as reported by parents or teachers. Again, levels of externalizing problems were very low in both family types in relation to the cutoff score for clinical problems. Neither were there differences between gay father and lesbian mother families for perceived stigma, quality of parenting, or parent–child interaction, reflecting low levels of perceived stigma, high levels of positive parenting low levels of negative parenting, and typical levels of parent–child interaction.

The ratings of internalizing and externalizing problems by the child psychiatrist, who was unaware of the child's family type, corroborated these findings. The 5% of children of gay fathers and 3.6% of children of lesbian mothers who showed a disorder are lower than the population norm for this measure (Meltzer, Gatward, Goodman, & Ford, 2000). Interestingly, the other studies of children born to gay fathers through surrogacy similarly found low levels of parent‐reported adjustment problems among the children (Baiocco et al., 2015; Green et al., 2015), especially in terms of internalizing problems (Green et al., 2015).

Although further examination of factors associated with variation in children's adjustment irrespective of family type showed neither parenting quality nor parents’ experience of stigmatization to be associated with children's internalizing problems as reported by parents and teachers, both of these factors predicted children's externalizing problems as reported by parents. Thus, as hypothesized, parents who perceived higher levels of stigma reported their children to show higher levels of externalizing problems. In addition, children who experienced higher levels of negative parenting were reported by their parents to show higher levels of externalizing problems. Both of these processes appeared to operate independently of each other as there was no association between negative parenting and perceived stigmatization. These findings are consistent with the large body of research showing negative parenting to be associated with children's externalizing problems in heterosexual parent families (Bornstein, 2002; Collins, Maccoby, Steinberg, Hetherington, & Bornstein, 2000). There is also a growing research literature showing that the stigmatization of gay and lesbian families is associated with externalizing problems in children (Bos & Gartrell, 2010; Bos & van Balen, 2008). No associations were identified between either parents’ experience of stigmatization or negative parenting and children's externalizing problems when reported independently by teachers. It is not known whether this reflected a difference in the perceptions of teachers or was due to the smaller sample of teachers.

A limitation of the study was the moderate sample size, which may have resulted in differences between the gay father and lesbian mother families not being detected. Although not all of the interview variables showed interrater agreement of .80, the coding of those that did not reach this threshold involved the use of nonverbal cues such as facial expression and gestures that were not available to the second rater. Thus, the interrater reliabilities of these interview variables may be underestimates. Although some of the assessments were carried out by Skype, there were no differences in any of the measures between families assessed in person and by Skype.

A further limitation was the use of volunteer samples. Although it was not possible to obtain a representative sample of gay father families, a variety of recruitment procedures were used to access as diverse a sample as possible. It should be emphasized that this is the first controlled, in‐depth study worldwide of parenting and child adjustment in the small but growing number of gay father families created through surrogacy and, as such, provides much needed data on the well‐being of children in this emerging family form. As the present study did not include a heterosexual comparison group, firm conclusions cannot be drawn regarding the absence of differences between gay father families formed through surrogacy and heterosexual parent families.

An advantage of the study was that data were obtained using a multimethod and multi‐informant approach. Although only 50% of the children's teachers completed the SDQ, significant correlations were obtained between parents’ and teachers’ SDQ scores for both externalizing (*r* = .55, *p* < .001) and internalizing problems (*r* = .40, *p* < .01), providing validation of the parents’ reports of their children's psychological adjustment. In addition, there were no differences in parent‐rated externalizing or internalizing SDQ scores between children whose teachers had and had not completed the SDQ. As some parents did not give consent for their children's teachers to be sent the questionnaire, the teachers’ actual response rate was 70%.

It cannot be ruled out that the lower levels of internalizing problems reported for the children of gay fathers resulted from the gay fathers being less aware of their children's internalizing problems than were the lesbian mothers. Externalizing problems may have been just as apparent to gay fathers as to lesbian mothers, as these tend to be more overt. Studies of heterosexual parent families show lower levels of parental sensitivity to children by fathers than mothers (Kwon, Jeon, Lewsader, & Elicker, 2012; Schoppe‐Sullivan et al., 2006) and may reflect differences in the ways in which men and women are socialized to parent (Fagan et al., 2014). Furthermore, the lower levels of parent‐reported internalizing problems among the children of gay fathers may have resulted from the higher incomes of gay fathers. Although there was no significant association between family income and children's internalizing problems when the relationship between the two was examined separately for gay father and lesbian mother families, when children's internalizing problems were residualized by income, there was no difference in children's internalizing problems between family types. Due to the high correlation between income and family type, it was not possible to fully disentangle the influence of family income from the influence of family type on children's internalizing problems. To the extent that there is a genetic component to children's development of internalizing problems (Gregory & Eley, 2007), it may be relevant that egg donors are screened for emotional disorders.

Overall, the study found the children of both gay fathers and lesbian mothers to show high levels of psychological adjustment and to have positive relationships with their parents. Stigmatization of the family and negative parenting were associated with higher levels of children's behavioral problems in both family types. These findings are consistent with the theoretical framework of the study (Overton, 2015) that emphasizes the bidirectional nature of relations between the social environment, parenting, and child adjustment. Research on gay father families formed through surrogacy is of interest in its own right as it is important to understand the psychological consequences for children of being conceived using the egg of a donor, born to a surrogate mother, and raised by two fathers, one of whom lacks a genetic connection to the child. However, this research is also of broader theoretical interest. By controlling for the presence of two parents in the family and the use of assisted reproduction, the study enabled the influence of parental gender on child development to be examined. The findings are consistent with those from studies of adoptive gay fathers (Farr et al., 2010a, 2010b; Farr & Patterson, 2013; Goldberg & Smith, 2013; Golombok et al., 2014) showing that men can be just as competent at parenting as women and that the absence of a female parent does not necessarily have adverse consequences for children's psychological adjustment. In addition, the finding that stigmatization and negative parenting were associated with higher levels of parent‐reported externalizing problems in children, irrespective of family type, contributes to the growing body of evidence that social and family processes are more influential in child adjustment than are structural variables, such as the gender and sexual orientation of parents.
